# Dietary Habits of Elite Soccer Players: Variations According to Competitive Level, Playing Position and Sex

**DOI:** 10.3390/nu15204323

**Published:** 2023-10-10

**Authors:** Jaime Sebastiá-Rico, Jose M. Soriano, Jesús Sanchis-Chordà, Miguel Alonso-Calvar, Pedro López-Mateu, David Romero-García, José Miguel Martínez-Sanz

**Affiliations:** 1 Area of Nutrition, University Clinic of Nutrition, Physical Activity and Physiotherapy (CUNAFF), Lluís Alcanyís Foundation—University of Valencia, 46020 Valencia, Spain; jaime.sebastia@fundacions.uv.es; 2 Food and Nutrition Research Group (ALINUT), University of Alicante, 03690 Alicante, Spain; 3 Food & Health Lab, Institute of Materials Science, University of Valencia, 46980 Paterna, Spain; 4 Joint Research Unit of Endocrinology, Nutrition and Clinical Dietetics, Health Research Institute La Fe—University of Valencia, 46026 Valencia, Spain; 5 Area of Nutrition, Academia Valencia CF SAD, 46980 Paterna, Spain; jsanchis@valenciacf.es; 6 Area of High Conditional Performance, Academia Valencia CF SAD, 46980 Paterna, Spain; 7 Area of Medical Services, Academia Valencia CF SAD, 46980 Paterna, Spain; 8 Nursing Department, Faculty of Health Sciences, University of Alicante, 03690 Alicante, Spain

**Keywords:** proteins, football, food, soccer, sport nutrition, carbohydrates, dietary fats

## Abstract

Soccer is a sport practiced worldwide by both men and women, where nutrition plays a fundamental role in the performance of soccer players, providing them with the nutrients necessary for energy, muscle recovery and injury prevention. The aim of this study is to describe the dietary habits in elite soccer players and their association with their competitive level, playing position and sex. A descriptive and non-experimental comparative study was conducted during the 2021–2022 competitive season. A total of 105 players belonging to a Spanish elite soccer team completed a food frequency questionnaire (FCFQ). It was observed that male players presented a higher consumption of carbohydrate-rich foods (*p* < 0.05), fermented foods (*p* = 0.014), frozen foods (*p* = 0.049) and red meat (*p* = 0.012) compared to female players, with the exception of lean meats, which were higher in females (*p* = 0.012). Furthermore, the U16-15 categories stand out for consuming carbohydrate-rich foods such as pasta (*p* = 0.000), bread (*p* = 0.004) and sweets (*p* = 0.046), as well as frozen foods (*p* = 0.002). Finally, alcohol consumption is higher in the senior categories (42.9%), where men are more likely to drink mixed drinks (6.2%), and beer and wine by women (10.7%). Practically no differences were found between the playing positions. In conclusion, differences were found in FCFQ according to competitive level and sex.

## 1. Introduction

Soccer is a team sport played by men and women in constant evolution, as it has experienced an increase in the physical and technical demands, as well as increased economic implications of winning or losing [1]. It consists of 22 male or female players on the field, grouped into two teams of 11 members, that confront each other with the aim of scoring a goal [2]. During training sessions and matches, players engage in a wide range of activities, which encompass both low- and high-intensity efforts. These activities include intermittent exercises of extended duration, such as walking, jogging, running at various speeds (both high and low), sprinting, moving in reverse, kicking, jumping, and tackling [1]. Moreover, studies have determined that, on average, players exhibit an oxygen uptake of approximately 70% of their maximum capacity during a match, with heart rates typically maintained at around 85% of their maximum levels [1,3]. There are two main categories: the base and the professional one, with the base category (U19-10) being the one played by young people before reaching the professional leagues (1st and 2nd in Spain) [4]. It is well known that body composition [5,6,7], as well as hydration, healthy eating habits and supplementation [3,8,9], are associated with physical performance among elite athletes. Understanding how these factors, along with playing position, may influence the nutritional intake of soccer players is essential for the design of nutrition education programs.

A study that analyzed the distance covered by soccer players found that wide defenders, central midfielders and wide midfielders covered a greater distance than center forwards, meanwhile the distance covered by center forwards was greater than central defenders’ distance [10]. Moreover, the intensity used to cover this distance also changes from one position to another, with the wide defenders, center forwards and wide midfielders being the ones who performed the greatest number of runs at very high intensity [11]. In addition, it has recently been observed that, depending on the playing position, the soccer player shows different body composition characteristics [7]. These results imply that individual differences in playing style need to be taken into account when planning nutritional and training strategies [12].

As mentioned above, nutrition plays a huge role in providing athletes the energy they require to meet their physical demands [3,8]. In addition, young athletes are in a phase of growth, development and dealing with body changes [13,14], which can be a great opportunity to promote healthy behaviors towards physical activity and diet, since these behaviors will continue into adulthood and impact long-term health and weight [15,16]. A few studies [17,18,19] that assessed the eating habits of professional male and female soccer players found that their nutrition intake was inadequate to sustain optimized performance throughout training and match play. This meta-analysis [20] gave an insight into the development of a macronutrient diet in senior and junior soccer players, finding a higher protein intake than the recommended and a carbohydrates (CHO) intake below the recommendations.

This other study that assessed the eating habits of junior soccer players showed a deficit in nutrient-rich foods, especially vegetables and fruits, and an excessive consumption of low-nutrient foods, highlighting above all the consumption of alcoholic beverages and soft drinks [21]. Those are valid reasons to think that nutrition education interventions are needed in team sports [22], since it has been shown that increasing an athlete’s nutrition knowledge can optimize physical performance [19,23,24] and lead to better dietary behaviors [25]. In this matter, there are several intervention tools that can be used to promote healthy eating behaviors in soccer players (i.e., posters, web apps, through activities), but the combination of a poster and a web app seems to be the most practical one in terms of providing the right information and helping them maintain those habits [26].

Lastly, the COVID-19 pandemic affected both daily training and absence from competitions, highlighting the importance of adjusting players’ dietary habits and eating patterns in order to preserve both their optimal health and performance in a context characterized by constant change [27,28].

Despite the fact this sport is widely practiced and accepted in our society, there is limited evidence about the eating habits of minors who play soccer and further differentiating between males and females. Therefore, the aim of this study is to describe the eating habits in elite soccer players and their association with their competitive level, playing position and sex. Accordingly, the following was initially hypothesized:

**Hypothesis** **(H1).** *Dietary habits will be similar among the different playing positions.*

**Hypothesis** **(H2).** *Dietary quality will be worse in the lower categories compared to the senior categories.*

**Hypothesis** **(H3).** *Men will consume more CHO, while women will consume more foods rich in healthy fats and lean proteins.*

## 2. Materials and Methods

### 2.1. Type of Study

This is a descriptive, cross-sectional and non-experimental study of dietary habits in elite soccer players of both sexes belonging to the Valencia C.F. Academy. The assessment was made in the month of May during the competitive season 2021–2022. The sample size calculation was performed with Rstudio software (version 3.15.0, Rstudio Inc., Boston, MA, USA). The significance level was set a priori at *p* = 0.05. The standard deviation (SD) was set according to the total SS data from previous studies on elite Spanish athletes (DE = 2.1) [29]. With an estimated error (d) of 0.49, the sample size needed was 70 athletes. The study population was selected by non-probabilistic, non-injury, convenience sampling among elite soccer players of both sexes belonging to the Valencia C.F. Academy.

### 2.2. Participants

A total of 100% of the Valencia Mestalla (n = 21), Juvenil A (n = 22), Juvenil B (n = 23), Cadete A (n = 32), Cadete Fundacions (n = 33), Valencia CF Femenino (n = 15) and Valencia CF Femenino B (n = 13) templates were included for the food consumption frequency questionnaire (FCFQ). However, as it was a voluntary questionnaire, it was finally completed by 110 of 159 soccer players (69.2%) (13 Valencia Mestalla players, 17 Juvenil A players, 16 Juvenil B players, 17 Cadete A players, 18 Cadete Fundacions players and all Valencia CF Femenino players (15) and Valencia CF Femenino B players (13)). All players had at least 4 years of soccer training experience and performed from 4 up to 7 regular training sessions per week (approximately 90 to more than 120 min per day), playing a theoretical official soccer match per week. The criteria for inclusion in this study were as follows: (a) be a healthy subject with medical authorization for the practice of federated sport; (b) belong to a team Valencia C.F. Academy; (c) being federated in soccer; (d) training a minimum of 4 days per week. The exclusion criteria for this study were as follows: (a) having been injured or having become ill during this study. Food portion size was estimated using photographs of a standardized portion, as well as an exact amount in grams or mL.

### 2.3. Procedure

In order to select the sample, the Valencia C.F. Academy sent a statement informing players of the execution of this study, instructions and inviting resident and non-resident players to collaborate. Before the players filled out/completed the questionnaire, subjects were informed about the purpose of this study. Informed consent was obtained and signed by those responsible for this study, as well as by the medical and coaching staff of the Valencia C.F. Academy. Each participant and their respective parents or legal guardians also signed it. The questionnaire was delivered electronically through a Google Form. The protocol complies with the Declaration of Helsinki for human research and is approved by the Ethics Committee of the University of Valencia (1534145).

### 2.4. Instruments

This study utilized a questionnaire that had been previously used in similar studies [21,30,31]. The selected questionnaire was validated for content, applicability, structure and presentation by University College Dublin and Crème Software Ltd., (Food4Me FFQ). It is a self-administered, online and semiquantitative food frequency questionnaire [32], with its Spanish version prepared by Bejar LM and collaborators [33]. It contains a total of 24 questions divided into two main sections. The first section collects the age, the team in which the subject plays (which identifies the subject’s sex) and the playing position. This section consists of 3 questions. The second section includes 21 questions that collated the consumption of several food and beverage groups over the previous month (average consumption of 157 food items): fruits, vegetables, pulses, white fish, blue fish, white meat, red meat, soft drinks and juices, sweets and ‘snacks’, ‘fast food’, and alcohol (beer, wine, mixed drinks, etc.). Frequency of consumption was measured by selecting one of the following options: never or less than once a month, 1–3 times a month, once a week, 2–4 times a week, 5–6 times per week, once a day, 2–3 times per day, 5–6 times per day, and >6 times per day.

The questionnaire can be found in the Supplementary Material section.

### 2.5. Statistical Analysis

The Kolmogorov–Smirnov normality test was performed to evaluate that all variables had a normal distribution. Kurtosis was also evaluated and Mauchly’s test of sphericity was performed to test the hypothesis of sphericity. Given the normal distribution of the data and with the aim of analyzing the frequency of food group and alcohol consumption, the chi-square test (χ^2^) was performed, segmenting the sample according to sex, category and playing positions. The minimum level of statistical significance was set at *p* < 0.05. All data were analyzed using the Statistical Package for the Social Sciences (SPSS) version 25.0 (IBM, Armonk, NY, USA).

## 3. Results

Table 1 shows the frequency of food consumption according to category. Significant differences were found for the consumption of pasta (*p* = 0.000), bread (*p* = 0.004), chicken and turkey (*p* = 0.042), eggs and egg products (*p* = 0.040), red meat (*p* = 0.035), sweets (*p* = 0.046) and prepared or frozen foods (*p* = 0.002). It was observed that cadets had a higher consumption of pasta, bread, sweets and prepared or frozen foods, but a lower consumption of chicken and turkey, while juveniles were the category with the highest consumption of eggs and their derivatives and red meat. In relation to the different playing positions, significant differences were only found for red meat consumption (*p* = 0.012), with a higher consumption by forwards.

Table 2 shows the frequency of food consumption according to sex. Significant differences were found for the consumption of fruits (*p* = 0.022), legumes (*p* = 0.001), pasta (*p* = 0.000), rice (*p* = 0.014), chicken and turkey (*p* = 0.012), red meat (*p* = 0.012), fermented foods (*p* = 0.014) and prepared or frozen foods (*p* = 0.049). In all these food groups, and with the exception of chicken and turkey consumption, it was observed that men had a higher consumption than women, while for chicken and turkey consumption, it was women who had a higher consumption.

Table 3 shows the frequency of alcohol consumption according to category. Significant differences were found for the question of whether or not they consumed alcohol (*p* = 0.000), showing that the frequency increased as the category increased, with the senior category consuming the most. Significant differences were also found for the type of alcohol consumed (*p* = 0.000), with juniors drinking more mixed drinks and seniors drinking more beer and wine. In relation to the different playing positions, significant differences were found for the question of whether or not they consumed alcohol (*p* = 0.028), with goalkeepers (30%) and midfielders (27.6%) consuming the most. Significant differences were also found for the type of alcohol consumed (*p* = 0.034), with beer being the most consumed by goalkeepers, and combined drinks and beer the most consumed by midfielders.

Table 4 shows the frequency of alcohol consumption according to sex. Significant differences were only found for the type of alcohol consumed (*p* = 0.024), with women consuming more beer and wine, and men consuming more mixed drinks.

## 4. Discussion

The aim of this study was to analyze the differences in the eating habits in elite soccer players by different competitive level, playing position and sex. To our knowledge, this is the first study analyzing these factors in elite soccer players from different level competitions, playing positions, and differentiating males and females. The main results showed a different food and alcohol intake according to the level of competition and sex.

### 4.1. Influence of Competitive Level on Nutrition

Dietary and physical exercise habits developed during the early years of life can have a lasting impact on long-term health and weight [34]. The nutritional approach aimed at young players faces the particular challenge of targeting individuals whose bodies undergo changes as they mature biologically, a process that does not always coincide with chronological age [3]. It was found that adolescents who were part of youth sports activities presented a greater tendency to consume fruits, vegetables and dairy products, although they also showed a greater inclination to ingest fast food and sugar-sweetened beverages compared to those who did not participate [35]. In addition, parents report poor availability of healthy food and beverage options at sporting events for youth athletes, contributing to the increased consumption of these products [36]. This was similar to our results, as the U16-15 were the soccer players who consumed the most sweets and frozen foods, with 8.6% of the subjects studied consuming both food groups between 3–4 times a week, and 54.3% and 51.4%, respectively, between 1–2 times a week. It is important to comply with the minimum recommendations for the frequency of consumption of some food groups such as ultra-processed foods because, although it has not been established for athletes as such, but for the general population, these are necessary in order to take care of health, physical recovery and body composition [3,37].

However, although there are previous studies highlighting a very low intake of healthy foods such as fruits and vegetables in youth stages, this has not been observed in our results, since in the case of the U16-15 category their daily consumption of one serving or more of fruits and vegetables was 62.8% and 28.6%, respectively, while consumption in the U19-17 categories was 54.3% and 37% [21]. Even so, it is true that there is still margin for improvement and it is necessary to continue applying food education so that all soccer players consume these foods on a daily basis, since fruits and vegetables are essential to obtain a correct supply of water, vitamins, minerals, antioxidants and fiber [3].

Moreover, the consumption of legumes was more frequent compared to other previous studies, where a frequency of 3–4 times a week or more was observed in the U16-15 categories of 31.5%, in the U19-17 categories of 39.1% and in the senior categories of 28.5% [21]. It is important to educate soccer players to consume not only animal protein, but also vegetable protein, especially from legumes, since they are very complete foods with a high contribution of CHO with a low glycemic index, fiber and micronutrients such as iron and vitamins, highlighting that in young ages, the contributions of these are superior in some cases compared to adults [3,30,38]. Regarding animal protein, it was abundant in lean meats, eggs and by-products and fish, with results similar to other previous studies [21,39,40].

In relation to foods rich in CHO such as cereals, whole grains, pasta, rice or tubers, it was high in all categories. There is increasing scientific evidence defending the benefits of high CHO intake compared to low CHO intake in preparation for competitions [3,20,41]. The reason is mainly based on the increase in muscle glycogen which provides (i) a greater endurance capacity in high-intensity exercises [42,43,44], (ii) an increase in the total distances covered [43,45], (iii) an improvement in specific soccer skills [42] or (iv) an increase in the time required until fatigue is reached [46]. However, it is important to apply CHO periodization during the week according to physical demands in order to properly maintain body composition and promote physiological adaptations to optimize sports performance [3,47].

### 4.2. Influence of Sex on Nutrition

Although women’s soccer has gained popularity and has experienced significant scientific advances, the amount of research on female players in this sport remains markedly lower compared to men’s soccer [3]. However, in the last decade, there has been a rapid increase in interest in women’s soccer, which has spurred the creation of better professional conditions for training and competition for female players [48,49].

In general, there could be a tendency for women to control their eating habits more than boys and to follow patterns that are considered healthier, both for health reasons and because of body dissatisfaction, at least in adolescence [50]. In a study that evaluated differences in dietary patterns between male and female soccer players, women showed significantly lower consumption of pork, bread, olive oil and soft drinks compared to men, and significantly higher consumption of seafood, natural fruit juice and fruit [30]. However, the lower consumption of red meat is the only similarity with our results, as women consumed less fruit among other differences. If we focus on micronutrients, it is worth highlighting the dietary needs of iron, where women require higher amounts (18 mg·d^−1^) than men (8 mg·d^−1^), being abundant in foods such as mollusks, red meat or legumes such as soybeans or lentils [3,51]. Our results highlighted that women consumed less red meat and legumes than men, so this could lead, if the athlete’s diet is not well structured through food or supplements, to suffering from pathologies such as anemia [3].

If we analyze the most relevant data on the frequency of consumption of the food groups, we observe a lower consumption of CHO (fruit, legumes, rice and pasta) by the female teams, which is in accordance with the literature reviewed in a study conducted with professional athletes who participated in several sports [52] and in another focused on male and female soccer players [30]. Although the reason for our female sample to consume less CHO is unknown, it could be due to misunderstandings about the impact of CHO intake on body composition, fear of weight gain and associated impacts on body image [53,54] and, for this reason, they tend to restrict foods high in this macronutrient.

In general, during prolonged submaximal aerobic exercise, women tend to rely more on fatty acids compared to men, especially when exercising at equivalent relative workload [55,56,57]. This observation could imply that women may require less CHO than male soccer players [58]. However, future research involving both female and male elite soccer players, matched under match and training conditions, is needed to determine whether energy substrate choice differs between genders. In fact, it is suggested that most female players may even minimize daily protein intake to prioritize higher carbohydrate intake for glycogen restoration, due to the low prevalence of their consumption [58].

Finally, in case the optimal intake of CHO through food is not reached, the use of CHO-rich sports foods such as gels, sports bars, gummies or isotonic drinks is recommended, as they can promote glycogen replenishment and improve performance, as observed in previous studies [3,59,60].

### 4.3. Influence of Playing Position on Nutrition

It has been seen that widefielder, fullback, center midfielder and center forward seem to be the most physically active playing positions during a match [61,62,63], which would mean that they are the most demanding in terms of energy expenditure and could explain the higher CHO requirements. Although there is a study where in some of these playing positions a higher consumption of CHO was observed, in our study, significant differences were only found for red meat consumption, with a higher consumption by forwards [12]. However, the questionnaire only includes the weekly frequency of the different food groups and not specific amounts of nutrients, so the results should not be misinterpreted. In addition, it should be remembered that the club has a dietician-nutritionist, so that nutrition, especially around training and matches, will be well advised.

Nutrient intake can have a profound impact on a player’s body composition and performance favoring a similarity with elite soccer patterns, where scientific literature advocates that each playing position shows specific characteristics due to different physical demands and roles during training and, especially, in competitions [3,6,7,64]. Therefore, it is recommended to take care of the nutrition of all players and, if possible, to individualize the nutrition based on the playing position.

### 4.4. Alcohol Consumption

There are multiple factors that are capable of interfering in the recovery of the soccer player, such as high muscle damage caused by eccentric movements (related to an inhibitory effect of muscle glycogen resynthesis in type II fibers [65]), sports injuries, poor sleep quality or excessive alcohol intake [3,66,67]. After training and/or matches, celebratory events and/or discouragement linked to the ingestion of alcohol in moderate or high amounts may occur. This is a practice that is recommended to be avoided as it seems to cause a reduction in the rate of myofibrillar protein synthesis (even if alcohol is ingested together with dietary protein), which may impair exercise adaptation and recovery by decreasing the anabolic responses of skeletal muscle [3]. Furthermore, let us not forget that it can produce dehydration in the soccer player in situations where rehydration is important for future physical demands (especially when there is more than one match per week) [3,67,68].

In our study, we observed that alcohol intake was higher as soccer players increased in age, which would be consistent due to the legality of consuming this type of beverages. Regarding the type of beverages, male players consumed mixed drinks more often than female players, which can also be seen in this study [30]. However, these authors observed a higher intake in older boys, while it comes from the youngest ones in our results. This could be due to the fact that younger boys have poorer nutritional knowledge, hence why it is important to intervene from an early age so that good decisions are maintained throughout the years [26]. Regardless of the type of beverage, it is advisable to consume the least amount of alcohol in order to ensure proper player health, as well as to optimize athletic performance, recovery from energy demands and body composition [3].

**Figure 1:**
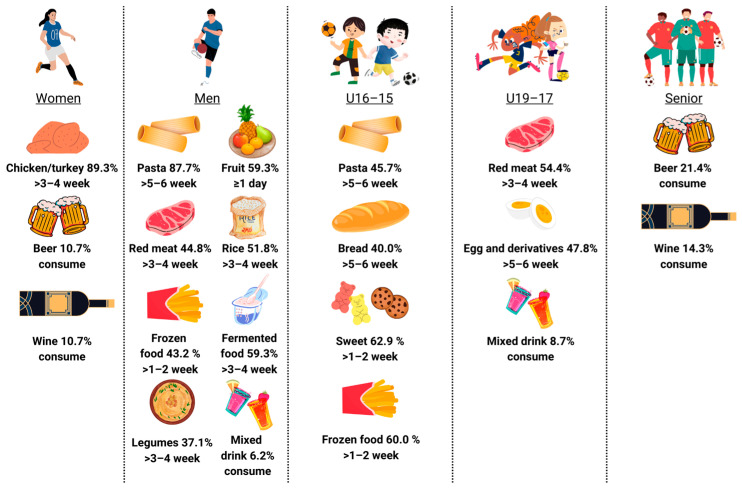
Most-consumed food and beverages classified by competitive level and gender.

For the first time to our knowledge, it was possible to assess the dietary habits of soccer players differentiated by competitive level, playing position and gender (Figure 1).

### 4.5. Limitations

This research had some limitations that should be discussed to improve its applicability to soccer sport contexts. Despite having a small study population, 100% of the Spanish soccer players of the selected sample were recruited, which is a significant sample size according to the statistical principles applied.

Using a food frequency questionnaire allows one to estimate the usual intake and is quick and easy to administer since it does not alter the habitual intake of the individual, it does not require trained interviewers, and it has a low administration cost, but the consumption information was collected in a self-reported and retrospective manner based on the memory of the soccer players. This could lead to errors in the number and type of foods declared. Although the questionnaire was useful to describe the eating habits of the sample, it is not accurate to quantify the portions eaten, sports supplements intake or the intake of vitamins and minerals. In addition, the actual intake of CHO, proteins and fats could not be quantified either. Despite this, this study has achieved its goal of describing the dietary habits and practices of elite soccer players, little studied previously. Another limitation was the sample; it was small in each category and there is heterogeneity between sex groups. Soccer players may change their eating habits over time, whether due to changes in their nutritional needs, professional recommendations or body composition goals among other reasons. The questionnaire may not capture these changes or require periodic updates to reflect changing consumption. Finally, the food frequency questionnaire used was a validated tool, although one of its limitations was that the information on consumption was collected in a self-reported and retrospective manner, based on the memory of the soccer players. In addition, they may have a tendency to respond in a socially desirable manner, which may influence the veracity of the information provided. However, in general, athletes tend to care about their diet and training, as their performance depends on it, and they tend to be knowledgeable about their eating habits. This might make them remember their food intake better compared to the general population.

It should be noted that the pandemic caused by COVID-19 may have influenced the eating habits of soccer players, but during the pandemic season (2020–2021 season), all soccer players had online counselling by the dietitian-nutritionist in order to avoid changing dietary habits from a healthy eating approach. In the 2021–2022 season, the operation of the club, picnics for matches, etc. functioned normally.

## 5. Conclusions

In conclusion, differences in the eating habits according to competitive level and sex were found in our sample. Men tend to consume more CHO-rich foods, fermented foods, frozen foods and red meat compared to women, while women frequent lean meats more than men. In terms of competitive levels, a higher intake of certain CHO-rich foods, in addition to unhealthy foods such as sweets and frozen foods, was observed in the lower categories. Practically no differences were found between the playing positions. In summary, it is recommended that daily consumption of alcohol, sweets and frozen food be reduced as much as possible, regardless of competitive level, playing position and gender.

## Figures and Tables

**Table 1:** Frequency (%) of food group consumption by soccer players of different competitive levels.

Items	Category (%)																		
	U16-15 (n = 35)						U19-17 (n = 46)						Senior (n = 28)						*p*
	<1 Week	1–2 Week	3–4 Week	5–6 Week	1–2 Day	≥3 Day	<1 Week	1–2 Week	3–4 Week	5–6 Week	1–2 Day	≥3 Day	<1 Week	1–2 Week	3–4 Week	5–6 Week	1–2 Day	≥3 Day	
Fruits	0.0	2.9	8.6	25.7	57.1	5.7	2.2	0.0	15.2	28.3	39.1	15.2	0.0	10.7	21.4	25.0	35.7	7.1	0.228
Vegetables	2.9	17.1	28.6	22.9	28.6	0.0	0.0	15.2	26.1	21.7	28.3	8.7	0.0	3.6	17.9	25.0	46.4	7.1	0.397
Vegetables rich in nitrates	28.6	22.9	31.4	17.1	0.0	0.0	19.6	28.3	28.3	13.0	10.9	0.0	21.4	35.7	32.1	3.6	7.1	0.0	0.441
Legumes	2.9	65.7	28.6	2.9	0.0	0.0	4.3	56.5	34.8	4.3	0.0	0.0	17.9	53.6	21.4	7.1	0.0	0.0	0.246
Tubers	2.9	34.3	48.6	14.3	0.0	0.0	4.3	23.9	45.7	21.7	4.3	0.0	3.6	21.4	50.0	17.9	7.1	0.0	0.834
Pasta	0.0	11.4	42.9	37.1	8.6	0.0	0.0	13.0	56.5	21.7	8.7	0.0	14.3	46.4	21.4	14.3	3.6	0.0	0.000
Rice	0.0	45.7	31.4	17.1	5.7	0.0	0.0	45.7	41.3	8.7	4.3	0.0	14.3	50.0	21.4	10.7	3.6	0.0	0.056
Bread	11.4	17.1	31.4	20.0	20.0	0.0	23.9	19.6	15.2	17.4	19.6	4.3	60.7	10.7	3.6	14.3	10.7	0.0	0.004
Whole grains	8.6	20.0	28.6	17.1	25.7	0.0	17.4	28.3	21.7	4.3	26.1	2.2	25.0	28.6	17.9	7.1	21.4	0.0	0.503
Chicken/turkey	0.0	22.9	45.7	17.1	14.3	0.0	0.0	15.2	39.1	41.3	4.3	0.0	7.1	7.1	53.6	21.4	10.7	0.0	0.042
Eggs and egg products	0.0	34.3	45.7	8.6	8.6	2.9	4.3	17.4	30.4	34.8	13.0	0.0	0.0	42.9	17.9	28.6	10.7	0.0	0.040
Fish	8.6	40.0	37.1	8.6	5.7	0.0	8.7	39.1	39.1	13.0	0.0	0.0	7.1	35.7	50.0	3.6	3.6	0.0	0.755
Red meat	8.6	45.7	34.3	2.9	8.6	0.0	6.5	39.1	41.3	10.9	2.2	0.0	25.9	37.0	25.9	0.0	3.7	7.4	0.035
Nuts	22.9	25.7	20.0	8.6	22.9	0.0	26.1	30.4	17.4	15.2	10.9	0.0	21.4	25.0	10.7	35.7	7.1	0.0	0.189
Fermented foods	2.9	37.1	20.0	28.6	11.4	0.0	8.7	34.8	17.4	26.1	13.0	0.0	25.0	21.4	35.7	10.7	7.1	0.0	0.070
Soft drinks	45.7	37.1	14.3	2.9	0.0	0.0	76.1	19.6	2.2	2.2	0.0	0.0	78.6	17.9	3.6	0.0	0.0	0.0	0.052
Pastries	37.1	54.3	8.6	0.0	0.0	0.0	67.4	30.4	2.2	0.0	0.0	0.0	57.1	42.9	0.0	0.0	0.0	0.0	0.046
Frozen foods	40.0	51.4	8.6	0.0	0.0	0.0	69.6	30.4	0.0	0.0	0.0	0.0	82.1	17.9	0.0	0.0	0.0	0.0	0.002

**Table 2:** Frequency (%) of food group consumption by male and female soccer players.

Items	Gender (%)												
	Women (n = 28)						Men (n = 81)						*p*
	<1 Week	1–2 Week	3–4 Week	5–6 Week	1–2 Day	≥3 Day	<1 Week	1–2 Week	3–4 Week	5–6 Week	1–2 Day	≥3 Day	
Fruits	3.6	10.7	25.0	21.4	28.6	10.7	0.0	1.2	11.1	28.4	49.4	9.9	0.022
Vegetables	0.0	7.1	32.1	17.9	32.1	10.7	1.2	14.8	22.2	24.7	33.3	3.7	0.491
Vegetables rich in nitrates	25.0	32.1	21.4	10.7	10.7	0.0	22.2	27.2	33.3	12.3	4.9	0.0	0.671
Legumes	21.4	53.6	14.3	10.7	0.0	0.0	2.5	60.5	34.6	2.5	0.0	0.0	0.001
Tubers	7.1	32.1	42.9	17.9	0.0	0.0	2.5	24.7	49.4	18.5	4.9	0.0	0.518
Pasta	14.3	42.9	25.0	14.3	3.6	0.0	0.0	13.6	49.4	28.4	8.6	0.0	0.000
Rice	14.3	42.9	32.1	7.1	3.6	0.0	0.0	48.1	33.3	13.6	4.9	0.0	0.014
Bread	50.0	17.9	7.1	14.3	10.7	0.0	22.2	16.0	21.0	18.5	19.8	2.5	0.085
Whole grains	25.0	28.6	25.0	3.6	17.9	0.0	13.6	24.7	22.2	11.1	27.2	1.2	0.521
Chicken/turkey	7.1	3.6	39.3	32.1	17.9	0.0	0.0	19.8	46.9	27.2	6.2	0.0	0.012
Eggs and egg products	3.6	28.6	28.6	25.0	14.3	0.0	1.2	29.6	33.3	24.7	9.9	1.2	0.914
Fish	7.1	46.4	39.3	3.6	3.6	0.0	8.6	35.8	42.0	11.1	2.5	0.0	0.720
Red meat	25.9	29.6	25.9	3.7	7.4	7.4	7.4	44.4	38.3	6.2	3.7	0.0	0.012
Nuts	32.1	32.1	10.7	14.3	10.7	0.0	21.0	25.9	18.5	19.8	14.8	0.0	0.603
Fermented foods	28.6	21.4	21.4	21.4	7.1	0.0	4.9	35.8	23.5	23.5	12.3	0.0	0.014
Soft drinks	82.1	17.9	0.0	0.0	0.0	0.0	61.7	27.2	8.6	2.5	0.0	0.0	0.162
Pastries	67.9	32.1	0.0	0.0	0.0	0.0	50.6	44.4	4.9	0.0	0.0	0.0	0.195
Frozen foods	82.1	17.9	0.0	0.0	0.0	0.0	56.8	39.5	3.7	0.0	0.0	0.0	0.049

**Table 3:** Frequency (%) of alcohol consumption by soccer players of different competitive levels.

Variables	Category (%)						Statistical Value
	U15-16 (n = 35)						
Alcoholic beverages	No			Yes			*p*
	100.0			0.0			0.000
Type	Nothing	Mixed drinks		Beer	Wine	Champagne	*p*
	100.0	0.0		0.0	0.0	0.0	0.000
Frequency	<1 week	1–2 week	3–4 week	5–6 week	1–2 day	≥3 day	*p*
	100.0	0.0	0.0	0.0	0.0	0.0	0.248
Variables	U19-17 (n = 46)						Statistical value
Alcoholic beverages	No			Yes			*p*
	89.1			10.9			0.000
Type	Nothing	Mixed drinks		Beer	Wine	Champagne	*p*
	89.1	8.7		2.2	0.0	0.0	0.000
Frequency	<1 week	1–2 week	3–4 week	5–6 week	1–2 day	≥3 day	*p*
	95.7	0.0	4.3	0.0	0.0	0.0	0.248
Variables	Senior (n = 28)						Statistical value
Alcoholic beverages	No			Yes			*p*
	57.1			42.9			0.000
Type	Nothing	Mixed drinks		Beer	Wine	Champagne	*p*
	57.1	3.6		21.4	14.3	3.6	0.000
Frequency	<1 week	1–2 week	3–4 week	5–6 week	1–2 day	≥3 day	*p*
	100.0	0.0	0.0	0.0	0.0	0.0	0.248

**Table 4:** Frequency (%) of alcohol consumption by male and female soccer players.

Variables	Gender (%)						Statistical Value
	Women (n = 28)						
Alcoholic beverages	No			Yes			*p*
	75.0			25.0			0.161
Type	Nothing	Mixed drinks		Beer	Wine	Champagne	*p*
	75.0	0.0		10.7	10.7	3.6	0.024
Frequency	<1 week	1–2 week	3–4 week	5–6 week	1–2 day	≥3 day	*p*
	100.0	0.0	0.0	0.0	0.0	0.0	0.401
Variables	Men (n = 81)						Statistical value
Alcoholic beverages	No			Yes			*p*
	87.7			12.3			0.161
Type	Nothing	Mixed drinks		Beer	Wine	Champagne	*p*
	87.7	6.2		4.9	1.2	0.0	0.024
Frequency	<1 week	1–2 week	3–4 week	5–6 week	1–2 day	≥3 day	*p*
	97.5	0.0	2.5	0.0	0.0	0.0	0.401

## Data Availability

The data presented in this study are available in the tables of this article. The data presented in this study are available on request from the corresponding author.

## Supplementary Materials

The following supporting information can be downloaded at the following: https://www.mdpi.com/article/10.3390/nu15204323/s1, File S1: Food consumption frequency questionnaire in elite soccer players.

## Abbreviations

CHO: Carbohydrates.

## References

[1] Bangsbo J., Mohr M., Krustrup P. (2006). Physical and Metabolic Demands of Training and Match-Play in the Elite Football Player. J. Sports Sci..

[2] Reglamentos|www.rfef.es. https://rfef.es/es/federacion/normativas-y-circulares/reglamentos.

[3] Collins J., Maughan R.J., Gleeson M., Bilsborough J., Jeukendrup A., Morton J.P., Phillips S.M., Armstrong L., Burke L.M., Close G.L. (2021). UEFA Expert Group Statement on Nutrition in Elite Football. Current Evidence to Inform Practical Recommendations and Guide Future Research. Br. J. Sports Med..

[4] Portada|www.rfef.es. https://rfef.es/es/portada.

[5] Högström G.M., Pietilä T., Nordström P., Nordström A. (2012). Body Composition and Performance: Influence of Sport and Gender among Adolescents. J. Strength Cond. Res..

[6] Sebastiá-Rico J., Soriano J.M., González-Gálvez N., Martínez-Sanz J.M. (2023). Body Composition of Male Professional Soccer Players Using Different Measurement Methods: A Systematic Review and Meta-Analysis. Nutrients.

[7] Sebastiá-Rico J., Martínez-Sanz J.M., González-Gálvez N., Soriano J.M. (2023). Differences in Body Composition between Playing Positions in Men’s Professional Soccer: A Systematic Review with Meta-Analysis. Appl. Sci..

[8] Kirkendall D.T. (1993). Effects of Nutrition on Performance in Soccer. Med. Sci. Sports Exerc..

[9] González-Neira M., San Mauro-Martín I., García-Angulo B., Fajardo D., Garicano-Vilar E. (2015). Valoración Nutricional, Evaluación de La Composición Corporal y Su Relación Con El Rendimiento Deportivo En Un Equipo de Fútbol Femenino. Rev. Esp. Nutr. Humana Dietética.

[10] Barros R.M.L., Misuta M.S., Menezes R.P., Figueroa P.J., Moura F.A., Cunha S.A., Anido R., Leite N.J. (2007). Analysis of the Distances Covered by First Division Brazilian Soccer Players Obtained with an Automatic Tracking Method. J. Sports Sci. Med..

[11] Rivilla-García J., Calvo L.C., Jiménez-Rubio S., Paredes-Hernández V., Muñoz A., van den Tillaar R., Navandar A. (2019). Characteristics of Very High Intensity Runs of Soccer Players in Relation to Their Playing Position and Playing Half in the 2013-14 Spanish La Liga Season. J. Hum. Kinet..

[12] Iglesias-Gutiérrez E., García A., García-Zapico P., Pérez-Landaluce J., Patterson A.M., García-Rovés P.M. (2012). Is There a Relationship between the Playing Position of Soccer Players and Their Food and Macronutrient Intake?. Appl. Physiol. Nutr. Metab. Physiol. Appl. Nutr. Metab..

[13] Villamor E., Jansen E.C. (2016). Nutritional Determinants of the Timing of Puberty. Annu. Rev. Public. Health.

[14] Smith J.W., Holmes M.E., McAllister M.J. (2015). Nutritional Considerations for Performance in Young Athletes. J. Sports Med..

[15] Hu T., Jacobs D.R., Larson N.I., Cutler G.J., Laska M.N., Neumark-Sztainer D. (2016). Higher Diet Quality in Adolescence and Dietary Improvements Are Related to Less Weight Gain During the Transition From Adolescence to Adulthood. J. Pediatr..

[16] Thomas D.T., Erdman K.A., Burke L.M. (2016). Position of the Academy of Nutrition and Dietetics, Dietitians of Canada, and the American College of Sports Medicine: Nutrition and Athletic Performance. J. Acad. Nutr. Diet..

[17] Russell M., Pennock A. (2011). Dietary Analysis of Young Professional Soccer Players for 1 Week during the Competitive Season. J. Strength Cond. Res..

[18] Briggs M.A., Cockburn E., Rumbold P.L.S., Rae G., Stevenson E.J., Russell M. (2015). Assessment of Energy Intake and Energy Expenditure of Male Adolescent Academy-Level Soccer Players during a Competitive Week. Nutrients.

[19] Devlin B.L., Leveritt M.D., Kingsley M., Belski R. (2017). Dietary Intake, Body Composition, and Nutrition Knowledge of Australian Football and Soccer Players: Implications for Sports Nutrition Professionals in Practice. Int. J. Sport. Nutr. Exerc. Metab..

[20] Steffl M., Kinkorova I., Kokstejn J., Petr M. (2019). Macronutrient Intake in Soccer Players-A Meta-Analysis. Nutrients.

[21] Cotán Cid J.D., Fernández de la Fuente A., Mata Ordóñez F., Sánchez-Oliver A.J. (2017). Análisis de la composición corporal y del consumo de alimentos y suplementos nutricionales en jugadores de división de honor juvenil de fútbol. EmásF Rev. Digit. Educ. Física.

[22] Sánchez-Díaz S., Yanci J., Castillo D., Scanlan A.T., Raya-González J. (2020). Effects of Nutrition Education Interventions in Team Sport Players. A Systematic Review. Nutrients.

[23] Ruiz F., Irazusta A., Gil S., Irazusta J., Casis L., Gil J. (2005). Nutritional Intake in Soccer Players of Different Ages. J. Sports Sci..

[24] Rossi F.E., Landreth A., Beam S., Jones T., Norton L., Cholewa J.M. (2017). The Effects of a Sports Nutrition Education Intervention on Nutritional Status, Sport Nutrition Knowledge, Body Composition, and Performance during Off Season Training in NCAA Division I Baseball Players. J. Sports Sci. Med..

[25] Valliant M.W., Emplaincourt H.P., Wenzel R.K., Garner B.H. (2012). Nutrition Education by a Registered Dietitian Improves Dietary Intake and Nutrition Knowledge of a NCAA Female Volleyball Team. Nutrients.

[26] Fernández-Álvarez M.M., Martín-Payo R., García-García R., Cuesta M., Carrasco-Santos S. (2020). A Nutrition Education Intervention in Adolescents Who Play Soccer: The IDEHA-F Project. Psicothema.

[27] Carter J.L., Kelly A.L., Williams R.A., Ford T.J., Cole M. (2021). Exploring Sports Nutritionists’ and Players’ Perspectives of Nutrition Practice within English Professional Football during the COVID-19 Pandemic. Sci. Med. Footb..

[28] Grazioli R., Loturco I., Baroni B.M., Oliveira G.S., Saciura V., Vanoni E., Dias R., Veeck F., Pinto R.S., Cadore E.L. (2020). Coronavirus Disease-19 Quarantine Is More Detrimental Than Traditional Off-Season on Physical Conditioning of Professional Soccer Players. J. Strength Cond. Res..

[29] Aguilar-Navarro M., Baltazar-Martins G., Brito de Souza D., Muñoz-Guerra J., Del Mar Plata M., Del Coso J. (2021). Gender Differences in Prevalence and Patterns of Dietary Supplement Use in Elite Athletes. Res. Q. Exerc. Sport..

[30] Soto-Célix M., Sánchez-Díaz S., Castillo D., Raya-González J., Domínguez-Díez M., Lago-Rodríguez Á., Rendo-Urteaga T. (2021). Consumo de alimentos, composición corporal y rendimiento físico en hombres y mujeres jóvenes jugadores de fútbol. Rev. Esp. Nutr. Humana Dietética.

[31] Ventura-Comes A., Martínez Sanz J.M., Sanchez-Oliver A., Domínguez R. (2019). Analysis of Foods Habits in Squash Players. J. Phys. Educ. Sport..

[32] Fallaize R., Forster H., Macready A.L., Walsh M.C., Mathers J.C., Brennan L., Gibney E.R., Gibney M.J., Lovegrove J.A. (2014). Online Dietary Intake Estimation: Reproducibility and Validity of the Food4Me Food Frequency Questionnaire against a 4-Day Weighed Food Record. J. Med. Internet Res..

[33] Bejar L.M., Sharp B.N., García-Perea M.D. (2016). The E-EPIDEMIOLOGY Mobile Phone App for Dietary Intake Assessment: Comparison with a Food Frequency Questionnaire. JMIR Res. Protoc..

[34] Manore M.M., Patton-Lopez M.M., Meng Y., Wong S.S. (2017). Sport Nutrition Knowledge, Behaviors and Beliefs of High School Soccer Players. Nutrients.

[35] Nelson T.F., Stovitz S.D., Thomas M., LaVoi N.M., Bauer K.W., Neumark-Sztainer D. (2011). Do Youth Sports Prevent Pediatric Obesity? A Systematic Review and Commentary. Curr. Sports Med. Rep..

[36] Thomas M., Nelson T.F., Harwood E., Neumark-Sztainer D. (2012). Exploring Parent Perceptions of the Food Environment in Youth Sport. J. Nutr. Educ. Behav..

[37] Avenue, 677 Huntington, Boston, Ma 02115 Healthy Eating Plate. https://www.hsph.harvard.edu/nutritionsource/healthy-eating-plate/.

[38] Abreu R., Figueiredo P., Beckert P., Marques J.P., Amorim S., Caetano C., Carvalho P., Sá C., Cotovio R., Cruz J. (2021). Portuguese Football Federation Consensus Statement 2020: Nutrition and Performance in Football. BMJ Open Sport Exerc. Med..

[39] Iglesias-Gutiérrez E., García-Rovés P.M., García A., Patterson A.M. (2008). Food Preferences Do Not Influence Adolescent High-Level Athletes’ Dietary Intake. Appetite.

[40] García-Rovés P.M., García-Zapico P., Patterson A.M., Iglesias-Gutiérrez E. (2014). Nutrient Intake and Food Habits of Soccer Players: Analyzing the Correlates of Eating Practice. Nutrients.

[41] Anderson L., Orme P., Naughton R.J., Close G.L., Milsom J., Rydings D., O’Boyle A., Di Michele R., Louis J., Hambly C. (2017). Energy Intake and Expenditure of Professional Soccer Players of the English Premier League: Evidence of Carbohydrate Periodization. Int. J. Sport. Nutr. Exerc. Metab..

[42] Baker L.B., Rollo I., Stein K.W., Jeukendrup A.E. (2015). Acute Effects of Carbohydrate Supplementation on Intermittent Sports Performance. Nutrients.

[43] Souglis A.G., Chryssanthopoulos C.I., Travlos A.K., Zorzou A.E., Gissis I.T., Papadopoulos C.N., Sotiropoulos A.A. (2013). The Effect of High vs. Low Carbohydrate Diets on Distances Covered in Soccer. J. Strength Cond. Res..

[44] Balsom P.D., Wood K., Olsson P., Ekblom B. (1999). Carbohydrate Intake and Multiple Sprint Sports: With Special Reference to Football (Soccer). Int. J. Sports Med..

[45] Bangsbo J., Nørregaard L., Thorsøe F. (1992). The Effect of Carbohydrate Diet on Intermittent Exercise Performance. Int. J. Sports Med..

[46] Goedecke J.H., White N.J., Chicktay W., Mahomed H., Durandt J., Lambert M.I. (2013). The Effect of Carbohydrate Ingestion on Performance during a Simulated Soccer Match. Nutrients.

[47] Anderson L., Drust B., Close G.L., Morton J.P. (2022). Physical Loading in Professional Soccer Players: Implications for Contemporary Guidelines to Encompass Carbohydrate Periodization. J. Sports Sci..

[48] Kirkendall D.T. (2020). Evolution of Soccer as a Research Topic. Prog. Cardiovasc. Dis..

[49] Nassis G.P., Brito J., Tomás R., Heiner-Møller K., Harder P., Kryger K.O., Krustrup P. (2022). Elite Women’s Football: Evolution and Challenges for the Years Ahead. Scand. J. Med. Sci. Sports.

[50] Bibiloni M.d.M., Pich J., Pons A., Tur J.A. (2013). Body Image and Eating Patterns among Adolescents. BMC Public Health.

[51] Office of Dietary Supplements—Iron. https://ods.od.nih.gov/factsheets/Iron-HealthProfessional/.

[52] Nunes C.L., Matias C.N., Santos D.A., Morgado J.P., Monteiro C.P., Sousa M., Minderico C.S., Rocha P.M., St-Onge M.-P., Sardinha L.B. (2018). Characterization and Comparison of Nutritional Intake between Preparatory and Competitive Phase of Highly Trained Athletes. Med. Kaunas. Lith..

[53] Carrigan K.W., Petrie T.A., Anderson C.M. (2015). To Weigh or Not to Weigh? Relation to Disordered Eating Attitudes and Behaviors Among Female Collegiate Athletes. J. Sport. Exerc. Psychol..

[54] McHaffie S.J., Langan-Evans C., Morehen J.C., Strauss J.A., Areta J.L., Rosimus C., Evans M., Elliott-Sale K.J., Cronin C.J., Morton J.P. (2022). Carbohydrate Fear, Skinfold Targets and Body Image Issues: A Qualitative Analysis of Player and Stakeholder Perceptions of the Nutrition Culture within Elite Female Soccer. Sci. Med. Footb..

[55] Tarnopolsky M.A. (2008). Sex Differences in Exercise Metabolism and the Role of 17-Beta Estradiol. Med. Sci. Sports Exerc..

[56] Roepstorff C., Thiele M., Hillig T., Pilegaard H., Richter E.A., Wojtaszewski J.F.P., Kiens B. (2006). Higher Skeletal Muscle alpha2AMPK Activation and Lower Energy Charge and Fat Oxidation in Men than in Women during Submaximal Exercise. J. Physiol..

[57] Lundsgaard A.-M., Kiens B. (2014). Gender Differences in Skeletal Muscle Substrate Metabolism—Molecular Mechanisms and Insulin Sensitivity. Front. Endocrinol..

[58] de Sousa M.V., Lundsgaard A.-M., Christensen P.M., Christensen L., Randers M.B., Mohr M., Nybo L., Kiens B., Fritzen A.M. (2022). Nutritional Optimization for Female Elite Football Players-Topical Review. Scand. J. Med. Sci. Sports.

[59] Russell M., Benton D., Kingsley M. (2012). Influence of Carbohydrate Supplementation on Skill Performance during a Soccer Match Simulation. J. Sci. Med. Sport..

[60] Sun F.-H., Cooper S.B., Chak-Fung Tse F. (2020). Effects of Different Solutions Consumed during Exercise on Cognitive Function of Male College Soccer Players. J. Exerc. Sci. Fit..

[61] Vigh-Larsen J.F., Dalgas U., Andersen T.B. (2018). Position-Specific Acceleration and Deceleration Profiles in Elite Youth and Senior Soccer Players. J. Strength Cond. Res..

[62] Pettersen S.A., Brenn T. (2019). Activity Profiles by Position in Youth Elite Soccer Players in Official Matches. Sports Med. Int. Open.

[63] Algroy E., Grendstad H., Riiser A., Nybakken T., Saeterbakken A.H., Andersen V., Gundersen H.S. (2021). Motion Analysis of Match Play in U14 Male Soccer Players and the Influence of Position, Competitive Level and Contextual Variables. Int. J. Environ. Res. Public. Health.

[64] Staśkiewicz W., Grochowska-Niedworok E., Zydek G., Białek-Dratwa A., Grajek M., Jaruga-Sȩkowska S., Kowalski O., Kardas M. (2022). Changes in Body Composition during the Macrocycle of Professional Football Players in Relation to Sports Nutrition Knowledge. Front. Nutr..

[65] Gunnarsson T.P., Bendiksen M., Bischoff R., Christensen P.M., Lesivig B., Madsen K., Stephens F., Greenhaff P., Krustrup P., Bangsbo J. (2013). Effect of Whey Protein- and Carbohydrate-Enriched Diet on Glycogen Resynthesis during the First 48 h after a Soccer Game. Scand. J. Med. Sci. Sports.

[66] Mujika I., Burke L.M. (2010). Nutrition in Team Sports. Ann. Nutr. Metab..

[67] Ranchordas M.K., Dawson J.T., Russell M. (2017). Practical Nutritional Recovery Strategies for Elite Soccer Players When Limited Time Separates Repeated Matches. J. Int. Soc. Sports Nutr..

[68] Heaton L.E., Davis J.K., Rawson E.S., Nuccio R.P., Witard O.C., Stein K.W., Baar K., Carter J.M., Baker L.B. (2017). Selected In-Season Nutritional Strategies to Enhance Recovery for Team Sport Athletes: A Practical Overview. Sports Med. Auckl. NZ.

