# Supplementary material for: Dietary Habits of Elite Soccer Players: Variations According to Competitive Level, Playing Position and Sex
Source: Nutrients. 2023 Oct 10;15(20):4323. doi: 10.3390/nu15204323 (PMC10609682; doi:10.3390/nu15204323)
Supplement: Supplementary file 1 [file nutrients-15-04323-s001.zip › nutrients-2614946-supplementary.pdf]

Cuestionario de frecuencia de consumo de alimentos en futbolistas de élite

Estimad@ deportista, estamos realizando un estudio sobre la frecuencia de consumo de alimentos en futbolistas de élite. El objetivo de este trabajo es realizar un análisis descriptivo y comparativo de los hábitos alimentarios en futbolistas pertenecientes a los diferentes equipos del Valencia C.F. Su colaboración nos será de gran ayuda, estándole agradecidos enormemente al contestar a estas preguntas.

Su identidad permanecerá en el anonimato, y sólo se usarán los datos de manera estadística para conocer la realidad del consumo de suplementos nutricionales en fútbol. La Ley Orgánica de Protección de Datos será respetada en todo momento. Este estudio ha sido aprobado por el comité de ética de la Universidad de Valencia con número de registro 1534145.

CONSENTIMIENTO

- He sido informado/a de las características del Proyecto de Investigación titulado: “Valoración cineantropométrica, nutricional y consumo de suplementos en futbolistas de élite”.
- He leído la información del proyecto, y he podido formular las dudas que me han surgido al respecto. Considero que he entendido dicha información.
- Estoy informado/a de la posibilidad de retirarme en cualquier momento del estudio.
- Estoy informado del modo en que serán tratados mis datos.

En virtud de tales condiciones, consiento participar en este estudio. El consentimiento se manifiesta con la realización de la encuesta

No rellenar más de una inscripción por persona y escribir en MAYÚSCULAS todas las respuestas que requieran texto.

* Indica que la pregunta es obligatoria

1. ¿A qué equipo perteneces?

Marca solo un óvalo.

- Valencia Mestalla
- Juvenil A
- Juvenil B
- Cadete A
- Cadete Fundacions
- Valencia CF Femenino
- Valencia CF Femenino B
- Valencia CF Femenino C

DATOS INICIALES

2. AÑO DE NACIMIENTO *

Ejemplo: 7 de enero del 2019

3. POSICIÓN DE JUEGO (Solo una opción) *

Marca solo un óvalo.

- Portero
- Defensa Lateral
- Defensa Central
- Mediocentro defensivo
- Centrocampista
- Extremo
- Delantero central

Cuestionario Frecuencia Consumo de Alimentos

4. 1.¿Cuántas piezas de fruta has consumido en los últimos 28 días?(1 pieza = aproximadamente 100 g) (incluido zumos de fruta) (1 porción = Aprox. 200 ml) *

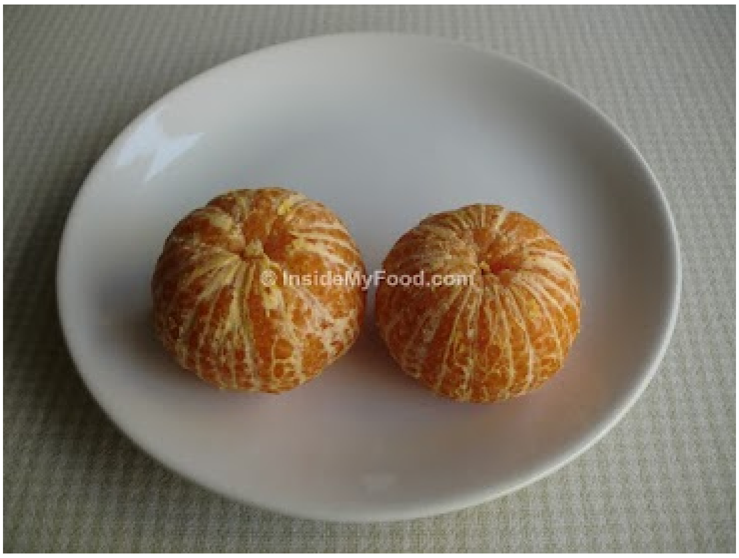

Marca solo un óvalo.

- Menos de una vez a la semana
- Una o dos veces a la semana
- 3-4 veces a la semana
- 5-6 Veces a la semana
- Una o dos veces al día
- 3 veces o más al día

5. 2.¿Cuántas porciones de verduras has consumido en los últimos 28 días? (1 porción = aproximadamente 150 g) *

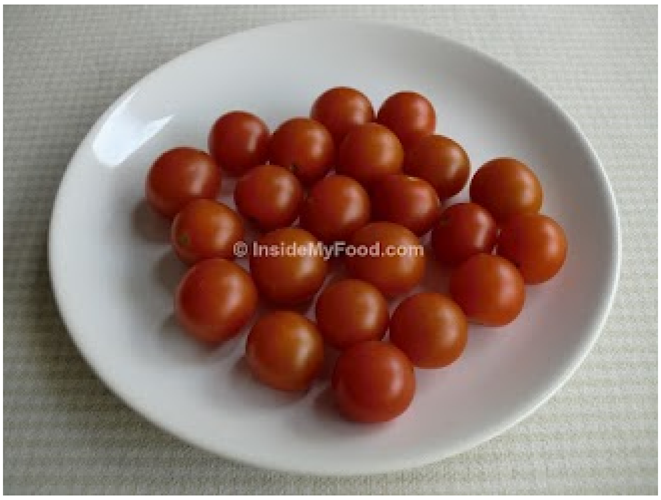

Marca solo un óvalo.

- Menos de una vez a la semana
- Una o dos veces a la semana
- 3-4 veces a la semana
- 5-6 Veces a la semana
- Una o dos veces al día
- 3 veces o más al día

6. 3.¿Cuántas porciones de las siguientes verduras has consumido en los últimos 28 días? (1 porción = aproximadamente 150 g): Apio, remolacha, espinaca, rúcula, endivia, puerro, rábano, pak choi/bok choy/col asiática *

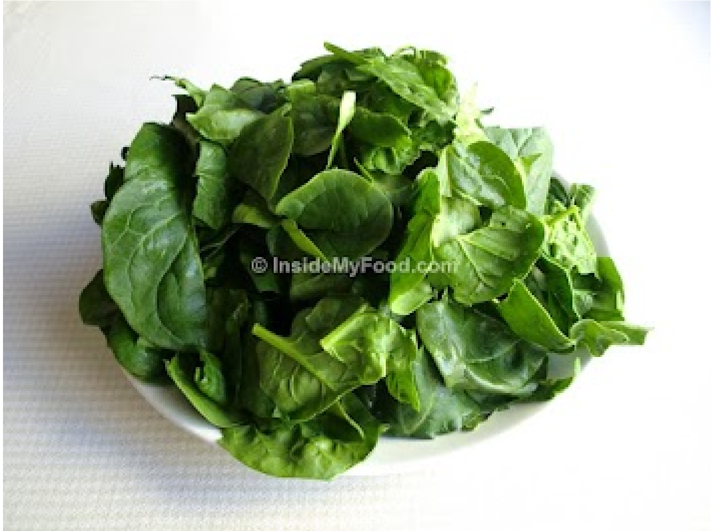

Marca solo un óvalo.

- Menos de una vez a la semana
- Una o dos veces a la semana
- 3-4 veces a la semana
- 5-6 Veces a la semana
- Una o dos veces al día
- 3 veces o más al día

7. 4. ¿Cuántas porciones de legumbres (lentejas, garbanzos, judías blancas, etc.) has consumido en los últimos 28 días? (1 porción = aprox. 60 g) *

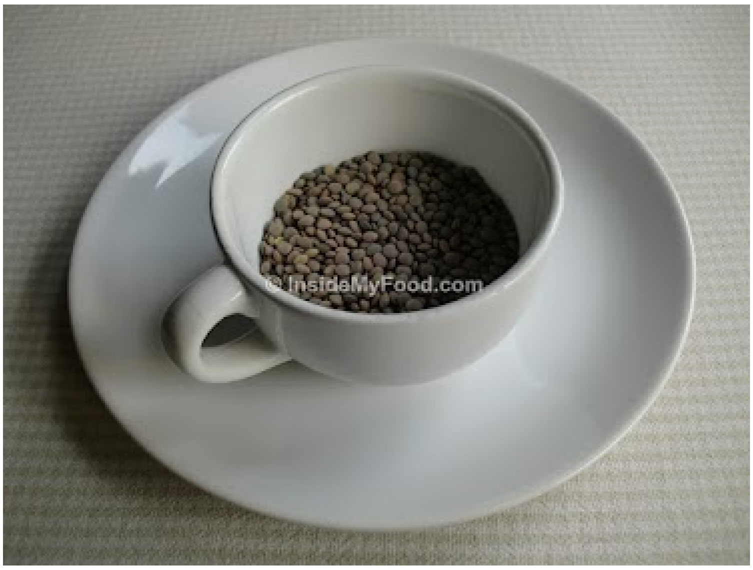

Marca solo un óvalo.

- Menos de una vez a la semana
- Una o dos veces a la semana
- 3-4 veces a la semana
- 5-6 Veces a la semana
- Una o dos veces al día
- 3 veces o más al día

8. 5. ¿Cuántas porciones de tubérculos (patata, boniato, yuca, etc.) has consumido en los últimos 28 días? (1 porción = aprox. 250 g) *

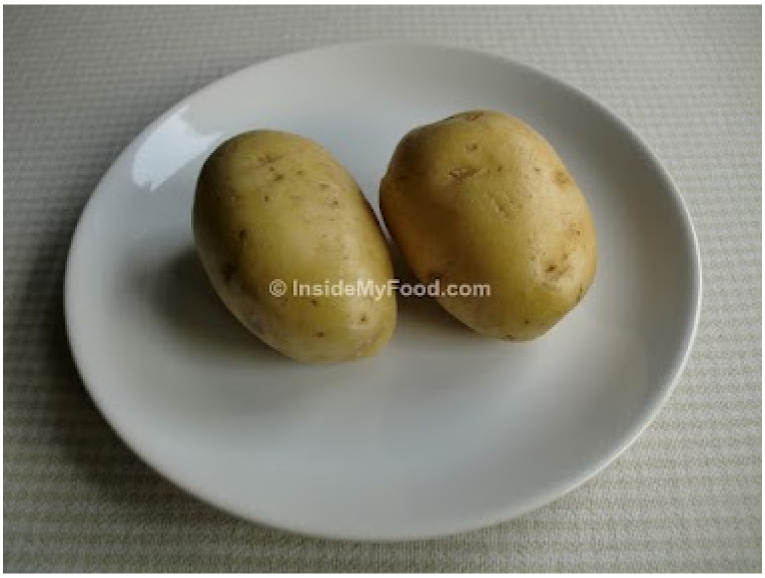

Marca solo un óvalo.

- Menos de una vez a la semana
- Una o dos veces a la semana
- 3-4 veces a la semana
- 5-6 Veces a la semana
- Una o dos veces al día
- 3 veces o más al día

9. 6. ¿Cuántas porciones de pasta (macarrón, espagueti, fideo, tallarín, canelón, etc.) has consumido en los últimos 28 días? (1 porción = aprox. 75 g) *

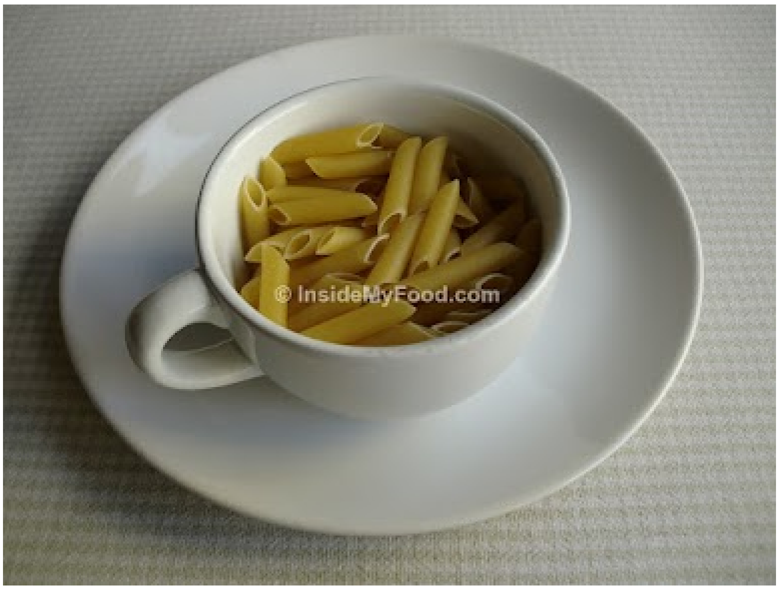

Marca solo un óvalo.

- Menos de una vez a la semana
- Una o dos veces a la semana
- 3-4 veces a la semana
- 5-6 Veces a la semana
- Una o dos veces al día
- 3 veces o más al día

10. 7. ¿Cuántas porciones de arroz has consumido en los últimos 28 dias? (1 porción = aprox. 75 g) *

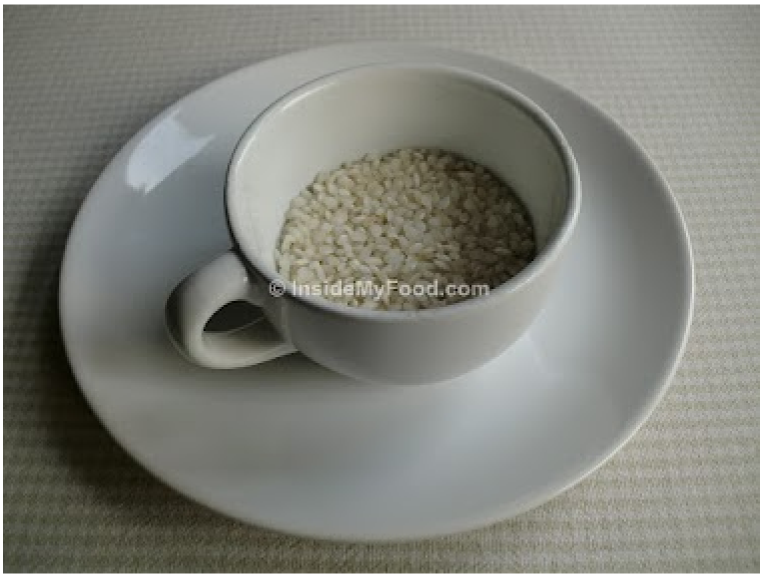

Marca solo un óvalo.

- Menos de una vez a la semana
- Una o dos veces a la semana
- 3-4 veces a la semana
- 5-6 Veces a la semana
- Una o dos veces al día
- 3 veces o más al día

11. 8. ¿Cuántas porciones de pan (molde, barra, tostado, etc.) has consumido en los últimos 28 dias? (1 porción = aprox. 50 g) *

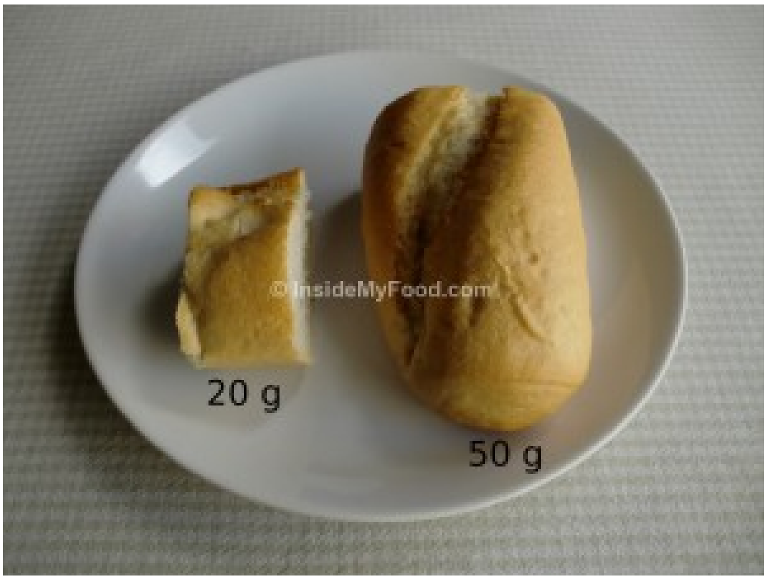

Marca solo un óvalo.

- Menos de una vez a la semana
- Una o dos veces a la semana
- 3-4 veces a la semana
- 5-6 Veces a la semana
- Una o dos veces al día
- 3 veces o más al día

12. 9. ¿Cuántas porciones de cereales integrales (pan integral, cereal tipo muesli, harina integral, etc.) has consumido en los últimos 28 dias? (1 porción = aprox. 50 g) *

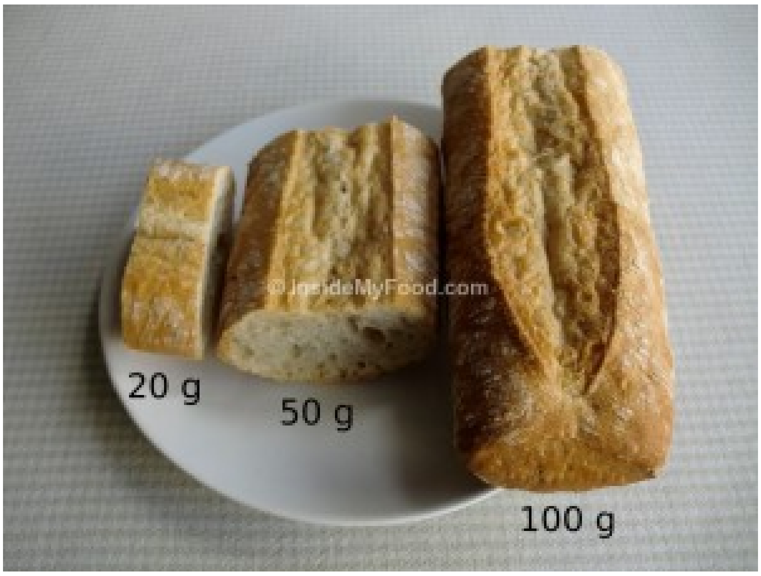

Marca solo un óvalo.

- Menos de una vez a la semana
- Una o dos veces a la semana
- 3-4 veces a la semana
- 5-6 Veces a la semana
- Una o dos veces al día
- 3 veces o más al día

13. 10. ¿Cuántas porciones de pollo / pavo has consumido en los últimos 28 días? *
(1 porción = aproximadamente 150 g)

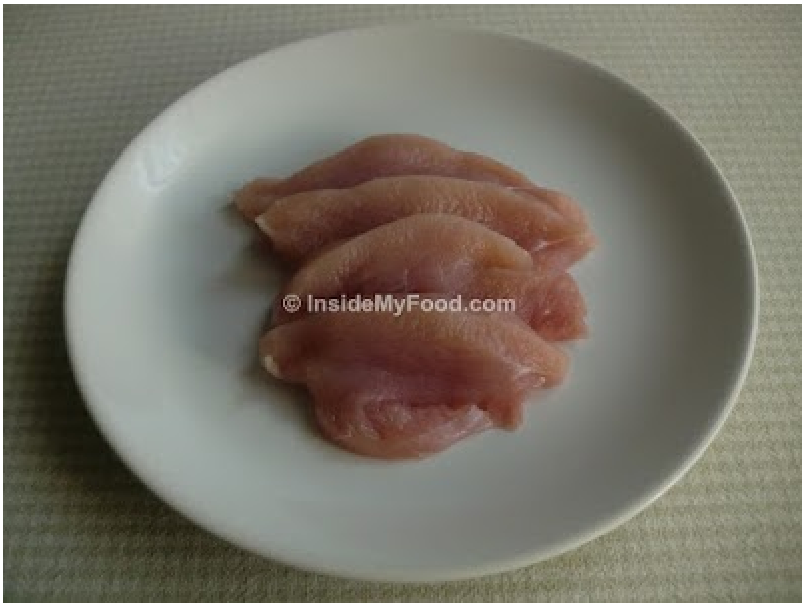

Marca solo un óvalo.

- Menos de una vez a la semana
- Una o dos veces a la semana
- 3-4 veces a la semana
- 5-6 Veces a la semana
- Una o dos veces al día
- 3 veces o más al día

14. 11. ¿Cuántas porciones de huevo y derivados has consumido en los últimos 28 días? (1 porción = aproximadamente 2 huevos de gallina) *

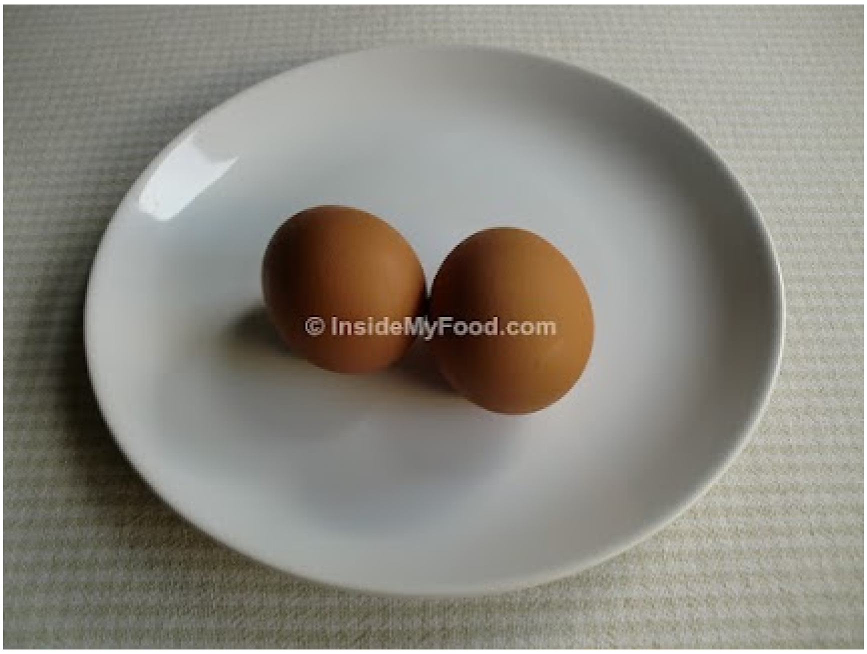

Marca solo un óvalo.

- Menos de una vez a la semana
- Una o dos veces a la semana
- 3-4 veces a la semana
- 5-6 Veces a la semana
- Una o dos veces al día
- 3 veces o más al día

15. 12. ¿Cuántas porciones de pescado has consumido en los últimos 28 días (1 porción = aproximadamente 150 g) *

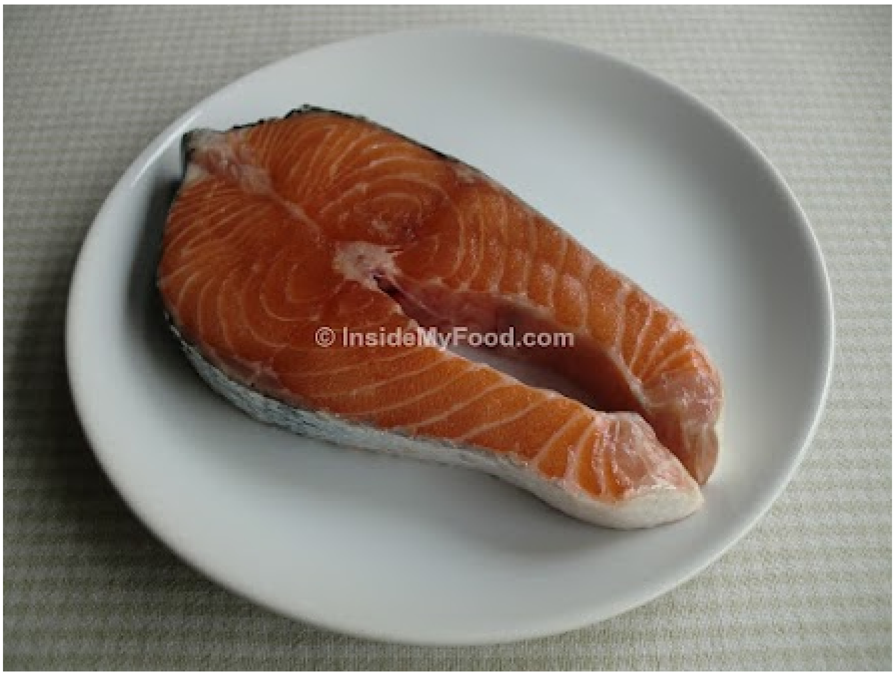

Marca solo un óvalo.

- Menos de una vez a la semana
- Una o dos veces a la semana
- 3-4 veces a la semana
- 5-6 Veces a la semana
- Una o dos veces al día
- 3 veces o más al día

16. 13. ¿Cuántas porciones de carne roja (carne, cerdo, cordero) has consumido en los últimos 28 días? (1 porción = aproximadamente 150 g)

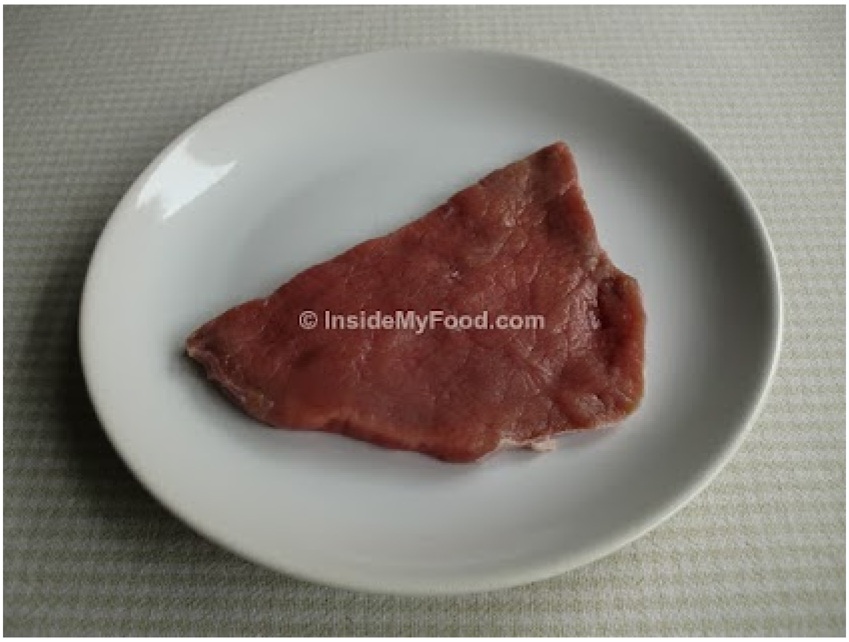

Marca solo un óvalo.

- Menos de una vez a la semana
- Una o dos veces a la semana
- 3-4 veces a la semana
- 5-6 Veces a la semana
- Una o dos veces al día
- 3 veces o más al día

17. 14. ¿Cuántas porciones de frutos secos (almendra, pistacho, nuez, etc.) has consumido en los últimos 28 días? (1 porción = aproximadamente 30 g pelados)

Imagen sin leyenda

Marca solo un óvalo.

- Menos de una vez a la semana
- Una o dos veces a la semana
- 3-4 veces a la semana
- 5-6 Veces a la semana
- Una o dos veces al día
- 3 veces o más al día

18. 15. ¿Cuántas porciones de alimentos fermentados (chucrut, kéfir, kombucha, kimchi, yogur natural, etc.) has consumido en los últimos 28 días? (1 porción = aproximadamente 125 g)

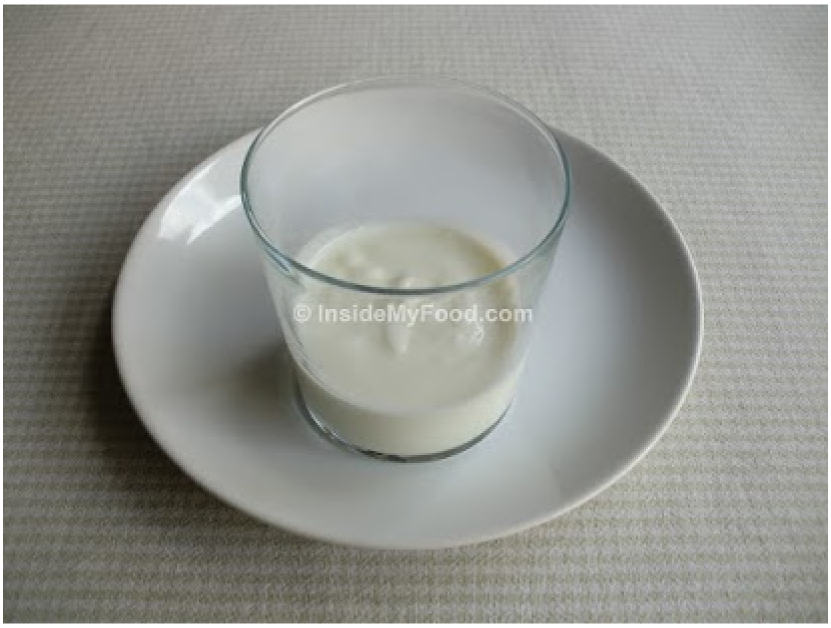

Marca solo un óvalo.

- Menos de una vez a la semana
- Una o dos veces a la semana
- 3-4 veces a la semana
- 5-6 Veces a la semana
- Una o dos veces al día
- 3 veces o más al día

19. 16. ¿Cuántas porciones de refrescos has consumido en los últimos 28 días? *
(1 porción = aproximadamente 250 ml)

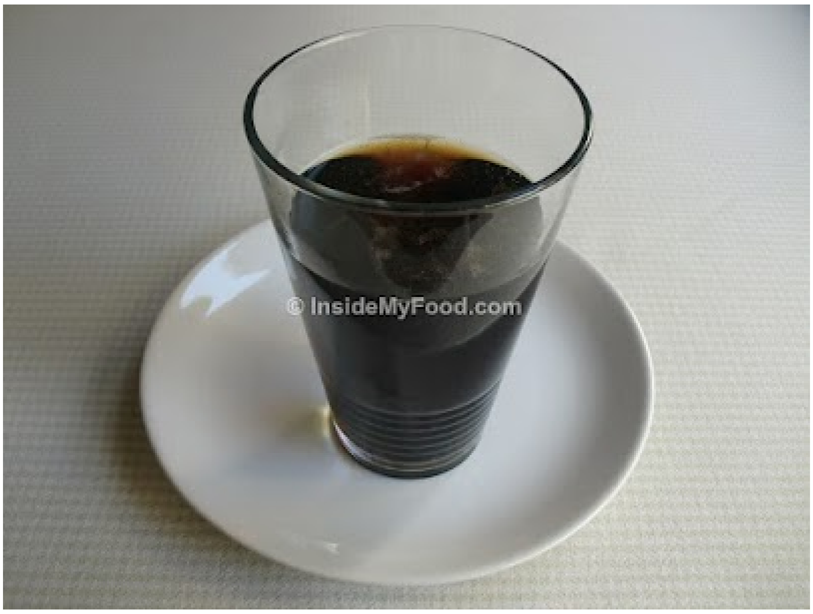

Marca solo un óvalo.

- Menos de una vez a la semana
- Una o dos veces a la semana
- 3-4 veces a la semana
- 5-6 Veces a la semana
- Una o dos veces al día
- 3 veces o más al día

20. 17. ¿Cuántas porciones de dulces producidos comercialmente (no hechos en casa) (Galletas / pasteles / bollería) has consumido en los últimos 28 días? (1 pieza = aproximadamente 100 g) *

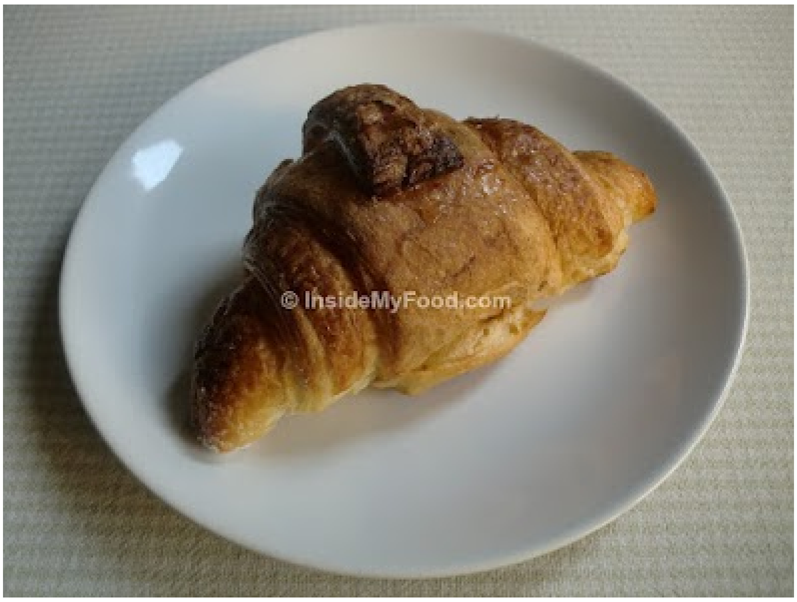

Marca solo un óvalo.

- Menos de una vez a la semana
- Una o dos veces a la semana
- 3-4 veces a la semana
- 5-6 Veces a la semana
- Una o dos veces al día
- 3 veces o más al día

21. 18. ¿Cuántas porciones de alimentos preparados / congelados has consumido (Croquetas, pizza, etc.) en los últimos 28 días? (1 porción = Aprox. 80 g) *

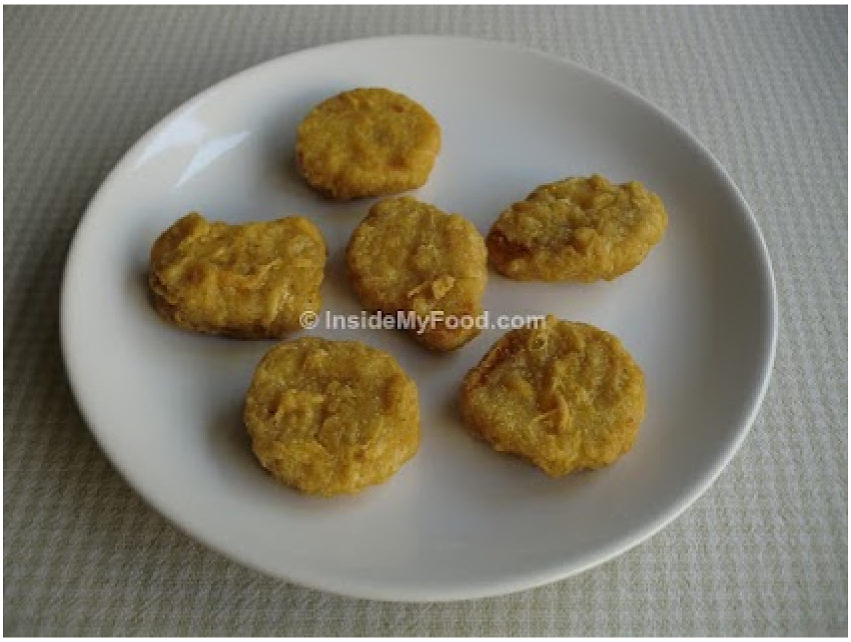

Marca solo un óvalo.

- Menos de una vez a la semana
- Una o dos veces a la semana
- 3-4 veces a la semana
- 5-6 Veces a la semana
- Una o dos veces al día
- 3 veces o más al día

22. 19. ¿Ha consumido bebidas alcohólicas en los últimos 28 días? *

Marca solo un óvalo.

- Sí
- No

23. 20. ¿Qué tipo de bebidas alcohólicas ha consumido en los últimos 28 días? *

Marca solo un óvalo.

- Cerveza
- Vino
- Bebidas mezcladas
- Otro: ____________________

24. 21. ¿Cuántas porciones de cerveza / vino / licores o bebidas mezcladas has consumido en los últimos 28 días? (1 porción de cerveza = aproximadamente 200 ml / 1Vaso de vino = aprox. 100 ml / 1 porción de licores o bebidas mezcladas =Aprox. 50 ml (de alcohol) *

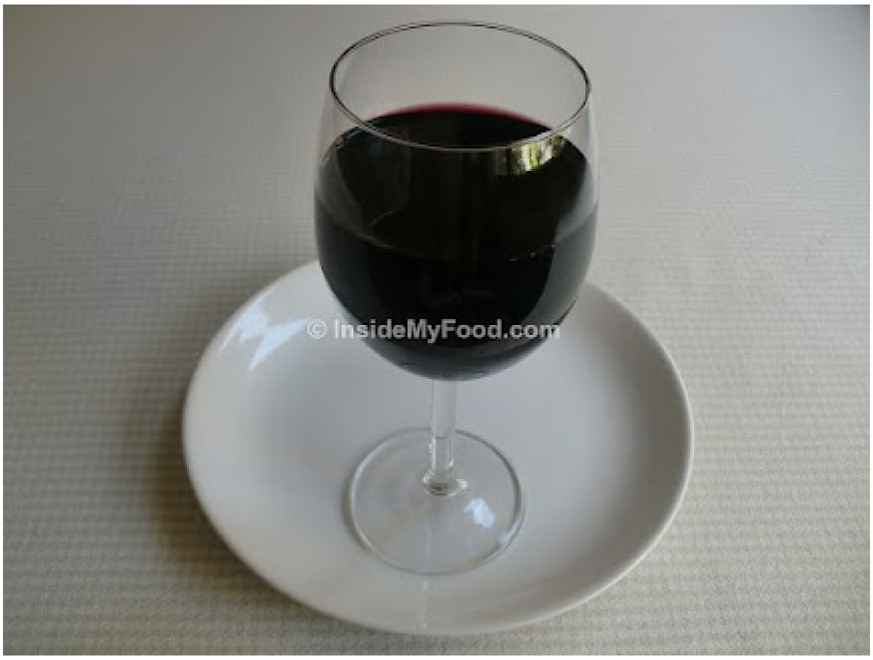

Marca solo un óvalo.

- Menos de una vez a la semana
- Una o dos veces a la semana
- 3-4 veces a la semana
- 5-6 Veces a la semana
- Una o dos veces al día
- 3 veces o más al día

Este contenido no ha sido creado ni aprobado por Google.

Google Formularios
